# Comparison of molecular diagnostic approaches for the detection and differentiation of the intestinal protist *Blastocystis* sp. in humans

**DOI:** 10.1051/parasite/2022029

**Published:** 2022-05-31

**Authors:** Martina Šloufová, Zuzana Lhotská, Milan Jirků, Klára J. Petrželková, C. Rune Stensvold, Ondřej Cinek, Kateřina Jirků Pomajbíková

**Affiliations:** 1 Institute of Parasitology, Biology Centre, the Czech Academy of Sciences České Budějovice 370 05 Czech Republic; 2 Department of Medical Biology, Faculty of Science, University of South Bohemia České Budějovice 370 05 Czech Republic; 3 Institute of Vertebrate Biology, Czech Academy of Sciences Květná 8 Brno 603 65 Czech Republic; 4 Department of Bacteria, Parasites and Fungi, Statens Serum Institut Copenhagen DK-2300 Denmark; 5 Department of Pediatrics, 2nd Faculty of Medicine, Charles University and Motol University Hospital Prague 150 06 Czech Republic

**Keywords:** *Blastocystis*, Conventional-PCR, qPCR, Sensitivity, Quantification, NGS

## Abstract

*Blastocystis* is the most commonly found intestinal protist in the world. Accurate detection and differentiation of *Blastocystis* including its subtypes (arguably species) are essential to understand its epidemiology and role in human health. We compared (i) the sensitivity of conventional PCR (cPCR) and qPCR in a set of 288 DNA samples obtained from stool samples of gut-healthy individuals, and (ii) subtype diversity as detected by next-generation sequencing (NGS) versus Sanger sequencing. Real-time PCR resulted in more positive samples than cPCR, revealing high fecal load of *Blastocystis* based on the quantification curve in most samples. In subtype detection, NGS was largely in agreement with Sanger sequencing but showed higher sensitivity for mixed subtype colonization within one host. This fact together with use of the combination of qPCR and NGS and obtaining information on the fecal protist load will be beneficial for epidemiological and surveillance studies.

## Abstract (fr)

*Blastocystis* est le protiste intestinal le plus répandu dans le monde. La détection et la différenciation précises de *Blastocystis*, y compris ses sous-types (sans doute des espèces), sont essentielles pour comprendre son épidémiologie et son rôle dans la santé humaine. Nous avons comparé (i) la sensibilité de la PCR conventionnelle (cPCR) et de la qPCR dans un ensemble de 288 échantillons d’ADN obtenus à partir d’échantillons de selles d’individus en bonne santé intestinale et (ii) la diversité des sous-types détectée par le séquençage de nouvelle génération (NGS) par rapport au séquençage Sanger. La PCR en temps réel a donné plus d’échantillons positifs que la cPCR, révélant une charge fécale élevée de *Blastocystis* sur la base de la courbe de quantification dans la plupart des échantillons. Dans la détection des sous-types, le NGS était largement en accord avec le séquençage de Sanger mais a montré une sensibilité plus élevée pour la colonisation de sous-types mixtes au sein d’un hôte. Ce fait, associé à l’utilisation de la combinaison de qPCR et de NGS et à l’obtention d’informations sur la charge fécale de protistes, sera bénéfique pour les études épidémiologiques et de surveillance.

## Introduction

*Blastocystis* sp. is a unicellular eukaryote colonizing the gastrointestinal tract of humans and various other species. Although discovered more than a century ago, its role in human health and disease has not been fully understood. Knowledge gaps remain in its epidemiology and interaction with the host, as well as factors affecting host colonization [3, 9, 26]. *Blastocystis* may be the most common intestinal human protist in the world, colonizing more than 1 billion people [1]. In some cohorts, the prevalence of *Blastocystis* sp. may reach 100% [5]. Based on small ribosomal subunit (SSU *rRNA*) gene analysis, at least 22 subtypes (ST) exist across mammalian and avian hosts [23]. Among these subtypes, ST1–ST9 and ST12 have been found in humans, with ST1–ST4 being commonly detected [26].

Despite the numerous surveys on *Blastocystis* sp., no consensus has been reached on the choice of method(s) for detection and differentiation of the protist (reviewed in Skotarczak [22]). Moreover, despite the development of molecular approaches, traditional microscopic examination of ova and parasites (O&P) and xenic culturing is still commonly used in laboratories to detect *Blastocystis* [12]. However, these methods require specialized technicians [12], are less sensitive, and do not provide subtype information [8, 24, 28]. Nevertheless, accurate detection and distinction of *Blastocystis* subtypes is essential to understand the transmission and the role of this protist in human health. Due to their high sensitivity and specificity, molecular methods such as conventional PCR (cPCR) or real-time PCR (qPCR) are often used [14, 22, 25]. In addition, next-generation sequencing (NGS) is gaining prominence in detection of *Blastocystis* and its subtypes [4, 17, 27].

The aim of this study was to compare (i) the sensitivity of cPCR and qPCR on a set of DNA samples obtained from stool samples of individuals with no gastrointestinal symptoms, and (ii) subtype diversity detected by cPCR and Sanger sequencing versus NGS.

## Material and methods

### Ethics statement

The studies involving human participants were reviewed and approved by Ethics Committee of the Biology Center of the Czech Academy of Sciences (reference number: 1/2017). Written informed consent to participate in this study was provided by the participants or their legal guardian/next of kin. All data were anonymized and processed according to valid laws of the Czech Republic (e.g., Act no. 101/2000 Coll and subsequent regulations). In case of the rat tissue used for testing of internal inhibition, we used samples from the experiment approved by the Committee on the Ethics of Animal Experiments of the Biology Centre of the Czech Academy of Sciences (České Budějovice, permit no. 33/2018) and by the Resort Committee of the Czech Academy of Sciences (Prague, Czech Republic) in strict accordance with Czech legislation (Act No. 166/1999 Coll. on veterinary care and on changes of some related laws, and Act No. 246/1992 Coll. on the protection of animals against cruelty), as well as the legislation of the European Union.

### Methods

In this study, we used 288 DNA samples obtained from fresh stool samples from a cohort created during a previous survey on the prevalence and diversity of *Blastocystis* in a gut-healthy human population in the Czech Republic (for more details on the collection and DNA extraction see Lhotská et al. [8]). We also used data on the positivity rate of *Blastocystis* sp. resulting from cPCR [8] for comparison with qPCR results obtained in the present study. Here, we applied the diagnostic qPCR protocol published in the study by Stensvold et al. [25]. The primers target the SSU rDNA fragment of 118 bp, which is detected by a Taqman probe. Samples were processed using a LightCycler LC 480 I (Roche, Basel, Switzerland) with a 96-well block. The cycling conditions consisted of primary denaturation (95 °C/10 min) and 37 × (95 °C/15 s, 60 °C/30 s, 72 °C/30 s). The results of qPCR on *Blastocystis* were then compared with the results of conventional PCR (from Lhotská et al. [8]) using McNemar’s test with Yates’s correction (0.5). Statistical analysis was performed using the software SciStatCalc 2013 (https://scistatcalc.blogspot.com/2013/11/mcnemars-test-calculator.html).

Positive samples from qPCR were subjected to amplicon NGS to determine *Blastocystis* subtypes: an informative fragment of SSU rDNA (~450 bp) was amplified, indexed and sequenced on a MiSeq instrument with the Reagent Kit v2, 2 × 250 bp (Illumina, San Diego, CA, USA); this was performed according to the method by Maloney et al. (2019) [11] with minor modifications in Cinek et al. [4] (for more detail see Supplementary data 1). These results were compared with the results on subtype diversity described in Lhotská et al. [8] based on Sanger sequencing. Fecal protist load was estimated based on a quantification curve generated from a dilution series of cultured *Blastocystis* ST3, which was set in the range of 10^0^ to 10^5^ cells per one qPCR reaction: 10^0^–10^1^ – mild fecal protist load; 10^2^–10^3^ – moderate fecal protist load; 10^4^–10^5^ – high fecal protist load (Supplementary data 2). *Blastocystis* cell counts from culture were calculated using a Bürker’s chamber and then serially diluted to obtain aliquots containing 10^0^, 10^1^, 10^2^, 10^3^, 10^4^, and 10^5^ cells, which were subsequently subjected to DNA extraction according to Lhotská et al. [8]. All negative samples were checked for PCR inhibition using addition of foreign DNA (obtained from tissue of experimental rats) and a specific qPCR protocol (commercial primers and Taqman probe for detection of the rat gene for beta-2 microglobulin; ThermoFisher Scientific, Waltham, MA, USA).

## Results

In this study, the prevalence of *Blastocystis* was determined by qPCR and subsequently compared with the results from cPCR obtained in our previous study Lhotská et al. [8]. In the set of 288 stool samples from the gut-healthy volunteers, the qPCR revealed a prevalence of 29% (83/288; Table 1) compared to cPCR with the prevalence 24% (71/288). Real-time PCR revealed 12 more positive samples (Table 1), our results indicate that qPCR is a more sensitive method for detecting *Blastocystis* in stool samples than cPCR (*p* < 0.05; *χ*^2^ = 8.26; Table 2). There was a discrepancy between these methods for two samples that qPCR evaluated as negative and cPCR as positive (Table 1). No internal inhibition was detected in any of the samples.

**Table 1:** Comparison of the sensitivity of conventional PCR (cPCR) and qPCR from the entire dataset of human samples (*n* = 288). Evaluation of the success of *Blastocystis* detection by next-generation sequencing (NGS) only in a set of qPCR-positive samples (*n* = 83).

Sample No.	Methods				Sample No.	Methods			
	cPCR	qPCR	Ct value	NGS		cPCR	qPCR	Ct value	NGS
B1	+	+	15	+	B2	+	+	19	+
B13	+	+	15	+	B19	+	+	19	+
B24	+	+	15	+	B115	+	+	19	+
B59	+	+	15	+	B126	+	+	19	+
B68	+	+	15	+	B184	+	+	19	+
B195	+	+	15	+	B220	+	+	19	+
B201	+	+	15	+	B374	+	+	19	+
B226	+	+	15	+	B417	+	+	19	+
B235	+	+	15	+	B86	+	+	20	+
B312	+	+	15	+	B292	+	+	20	+
B339	+	+	15	+	B277	+	+	21	−
B371	+	+	15	+	B303	+	+	21	+
B373	+	+	15	+	B380	+	+	21	+
B9	+	+	16	+	B300	+	+	22	+
B37	+	+	16	−	B375	+	+	22	+
B42	+	+	16	+	B418	+	+	22	+
B45	+	+	16	+	B424	+	+	22	+
B49	+	+	16	+	B431	+	+	23	+
B120	+	+	16	+	B33	+	+	24	+
B225	+	+	16	+	B36	+	+	24	+
B327	+	+	16	+	B313	+	+	24	+
B343	+	+	16	+	B365	+	+	24	+
B352	+	+	16	+	B55	+	+	26	+
B364	+	+	16	+	B144	−	+	28	+
B412	+	+	16	+	B345	+	+	28	+
B15	+	+	17	+	B405	+	+	29	+
B30	+	+	17	+	B356	+	+	30	−
B65	+	+	17	+	B372	−	+	31	+
B82	+	+	17	+	B10	−	+	32	−
B99	+	+	17	+	B35	−	+	32	−
B113	+	+	17	+	B38	−	+	32	−
B185	+	+	17	+	B41	−	+	32	+
B336	+	+	17	+	B50	−	+	32	−
B341	+	+	17	+	B54	−	+	32	−
B353	+	+	17	+	B62	−	+	32	−
B363	+	+	17	+	B114	−	+	32	+
B31	+	+	18	−	B189	−	+	32	−
B224	+	+	18	+	B240	−	+	32	+
B231	+	+	18	+	B248	−	+	32	−
B393	+	+	18	−	B398	−	+	32	−
B397	+	+	18	+	B425	+	+	32	+
B413	+	+	18	+					

**Table 2:** Comparison of results of qPCR (Stensvold et al., 2012) and conevntional PCR [cPCR] (Sciclune et al., 2006) in detection of *Blastocystis* sp. using McNemar test (*p* < 0.004; χ^2^ = 8.265).

	qPCR		
cPCR	Positive	Negative	
Positive	69	2	71 (25%)
Negative	14	203	217 (75%)
	83 (29%)	205 (71%)	288

We established a quantification curve (10^0^–10^5^ of cells/1 qPCR reaction) to evaluate the *Blastocystis* fecal load in positive samples and to extrapolate different colonization intensities from ct values (ct values are displayed for each sample in Table 1). In more than half of the samples positive in qPCR (52/83), colonization intensities reached 10^5^ or more, with the range of ct values ranging from 15 to 20 (Table 3). Fecal protist load 10^3^–10^4^ (range of ct values between 21 and 27) was found in 13 samples, and 10^1^–10^2^ (range of ct values between 28 and 32) in 18 samples (Table 3). In the samples positive only in qPCR (*n* = 12), a very low fecal protist load was found, i.e., 10^1^–10^2^ (Table 3).

**Table 3:** Evaluation of fecal load of *Blastocystis* in human samples based on the established quantification curve (set in the range of 10^0^ to 10^5^ cells per 1 qPCR reaction).

Estimated fecal protist load^1^	Number of samples/Number of positive samples	Ct value range
10^1^–10^2^	18/83	28–32
10^3^–10^4^	13/83	21–27
10^5^ –10^6^	52/83	15–20

1 Number of cells per 1 qPCR reaction.

Subtype diversity for all 83 qPCR-positive samples was evaluated by NGS, which detected subtypes in 69 samples (69/83; Tables 1 and 4). In case of the presence of one subtype in a sample, the NGS results were consistent with our previous results based on Sanger sequencing [8]. In fact, the major benefit of NGS appears to be in its ability to detect mixed colonizations of different subtypes in one sample. Mixed colonizations were found in five more cases compared to Sanger sequencing, specifically the subtype colonization mix: ST1 + ST7, ST1 + ST3, ST2 + ST3 (2×), ST3 + ST7 (Table 4). In the case of 12 samples positive only in qPCR with low fecal protist load, NGS detected subtypes in only five samples, namely ST2, ST5, ST3 (2×) and ST4 (Table 4).

**Table 4:** Comparison of *Blastocystis* subtype data in a set of 83 qPCR-positive samples obtained by Sanger sequencing (results obtained in previous study Lhotská et al., 2020) and next-generation sequencing (NGS).

Sample No.	Subtype		Sample No.	Subtype	
	Sanger sequencing	NGS		Sanger sequencing	NGS
B1	ST3	ST3	B225	ST1	ST1
B2	ST1	ST1	B226	ST1	ST1
B9	ST1	ST1	B231	ST3	ST3 + ST1
B10	–	–	B235	ST3	ST3
B13	ST1	ST1	B240	–	ST3
B15	ST3	ST3	B248	–	–
B19	ST3	ST3	B277	ST7	–
B24	ST6	ST6	B292	ST7	ST7
B30	ST3	ST3	B300	ST4	ST4
B31	ST3	–	B303	ST7	ST7
B33	ST3	ST3	B312	ST3	ST3
B35	–	–	B313	ST3	ST3
B36	ST1	ST1	B327	ST2	ST2
B37	ST2	–	B336	ST3	ST3
B38	–	–	B339	ST1	ST1
B41	–	ST3	B341	ST3	ST3
B42	ST1	ST1	B343	ST5	ST5
B45	ST1	ST1 + ST7	B345	ST6	ST6
B49	ST1	ST1	B352	ST3	ST3 + ST2
B50	–	–	B353	ST1 + ST3	ST1 + ST3
B54	–	–	B356	ST3	–
B55	ST3	ST3	B363	ST3	ST3
B59	ST4	ST4	B364	ST3	ST3 + ST2
B62	–	–	B365	ST7	ST7 + ST3
B65	ST4	ST4	B371	ST4	ST4
B68	ST3	ST3	B372	–	ST4
B82	ST2	ST2	B373	ST4	ST4
B86	ST3	ST3	B374	ST2	ST2
B99	ST3	ST3	B375	ST1	ST1
B113	ST2	ST2	B380	ST3	ST3
B114	–	ST2	B393	ST7	–
B115	ST7	ST7	B397	ST2	ST2
B120	ST1	ST1	B398	–	–
B126	ST6	ST6	B405	ST6	ST6
B144	–	ST5	B412	ST2	ST2
B184	ST3	ST3	B413	ST4	ST4
B185	ST6	ST6	B417	ST2	ST2
B189	–	–	B418	ST2	ST2
B195	ST3	ST3	B424	ST3	ST3
B201	ST3	ST3	B425	ST2	ST2
B220	ST3	ST3	B431	ST4	ST4
B224	ST1	ST1			

## Discussion

To compare the sensitivity between the two PCR-based approaches for detection of *Blastocystis*, we used a dataset of 288 human stool samples obtained in the study by Lhotská et al. [8]. Revealing 12 more positive samples, qPCR was the most sensitive method for detection of *Blastocystis*. The overall prevalence of *Blastocystis* by qPCR and cPCR was 29% and 24% (Lhotská et al. [8]), respectively. Surprisingly, it appears that this is the very first study comparing the sensitivity between the commonly used cPCR protocol [20] and qPCR [25] for the detection of *Blastocystis* sp. Previously, some studies showed higher sensitivity of qPCR in comparison with classical methods such as direct-light microscopy or xenic *in vitro* culture [14, 15, 25]. The study by Nourrisson et al. [14] compared four qPCR protocols for detection of *Blastocystis* sp. and found that they differed in specificity and sensitivity. Furthermore, the authors recommend the qPCR protocol Stensvold et al. [25] for diagnostic purposes and to add another method for subtype identification.

Despite higher sensitivity, qPCR scored two samples as negative, while conventional PCR scored them positive; these two samples were positive for ST3 and ST8. The two false-negative results by qPCR might be due to the degradation of DNA in the samples due to long-term storage and repeated freeze-thawing cycles of their aliquots. These DNA samples were tested again by cPCR, one sample appears to be negative and one (ST8) showed much less intensive amplicon in the electrophoresis. Alternatively, the qPCR protocol might have limited sensitivity for example for ST8, which was not used in the validation panel by Stensvold et al. [25], who developed the method. However, the applicability of the primers and probe was validated *in silico* using the alignment in the article’s Figure 1 [25] with a 100% match to ST8, which means that, at least in theory, the assay should be able to pick up this subtype. In addition, no inhibition was revealed in any sample during inhibition control using the foreign DNA.

The advantage of qPCR-based diagnostic approach is the ability to estimate the fecal load of *Blastocystis* in colonized humans based on an established quantification curve. Our results in individuals with healthy intestines (i.e., without inflammatory diseases) showed a high fecal *Blastocystis* load in more than half of the samples. This fecal load ranged in values of order from 10^5^ to 10^6^ cells per one qPCR reaction. In the 12 samples scored as positive only by qPCR, low fecal protist load was detected (10^1^–10^2^ cells per sample). A very recent study by Cinek et al. [4] quantified *Blastocystis* in feces of asymptomatic children and adolescents. However, more studies on both healthy humans and patients with inflammatory of functional bowel diseases are warranted [13]. A comparison of fecal *Blastocystis* loads between healthy and sick individuals could fundamentally contribute to understanding the role of *Blastocystis* sp. in the human gut ecosystem and could be important for experimental studies testing the effect of *Blastocystis* sp. on gut inflammation [2]. It is important to note that the quantification curve for assessing fecal *Blastocystis* load might be biased by different copy number of the SSU rRNA gene in individual subtypes and life stages of *Blastocystis*. This could slightly reduce the accuracy of quantification data. However, such data for *Blastocystis* and its subtypes are not yet available. Nevertheless, an approximate determination of *Blastocystis* fecal load can reveal trends between different human cohorts.

In epidemiological studies on *Blastocystis* sp. in humans, the identification of its subtypes plays an important role [6, 8, 16, 21]. Because different *Blastocystis* subtypes colonize different hosts and apparently differ in geographical distribution, surveys aimed at subtype determination might help reveal transmission pathways and potential sources of specific subtypes in a particular area. To date, most studies used Sanger sequencing for subtype identification [7, 8, 18], which may have limitations in detecting mixed subtype colonizations. Here, we subjected all 83 qPCR-positive samples to NGS analysis to determine subtypes. We found that subtype diversity was largely consistent with the results of Sanger sequencing by Lhotská et al. [8], in which Sanger sequencing was used. In 12 samples identified as positive only by qPCR, the NGS revealed subtypes only in five samples which was probably caused by low fecal load of *Blastocystis*. The remaining seven samples were confirmed by Sanger sequencing from qPCR amplicons (118 bp), however, without information about subtypes.

Although epidemiological studies usually describe colonization of an individual with only one subtype of *Blastocystis* sp. [8, 19, 21], mixed subtype colonization appears to be more common [17, 22, 29]. This situation is in part caused by limitations of some of the current molecular tools, which preferentially amplify the predominant subtypes present in a sample [11]. Here, the NGS-based approach showed higher sensitivity in determining mixed subtype colonization than a combination of methods, such as conventional PCR and Sanger sequencing (for more details see Lhotská et al. [8]). While Lhotská et al. [8] revealed a single case of mixed infection, NGS detected five more cases of mixed colonisation.

From a diagnostic point-of-view, our results support the fact that qPCR is the most suitable method for detecting the presence of *Blastocystis*. NGS alone cannot achieve qPCR sensitivity, mainly due to the known signal crosstalk between individual samples in a sequencing run (e.g., [10]). Although this issue can be alleviated by using unique dual indexing, it cannot be eliminated. Therefore, very low read counts do not necessarily indicate presence of the organism. Thus, the role of NGS in *Blastocystis* diagnostics is primarily in the determination of its subtypes and disentangling mixed colonizations. Of the 83 total qPCR-positive samples, the NGS revealed subtypes in 69 samples.

## Conclusion

To understand the epidemiology of *Blastocystis* sp., it is necessary to establish a gold standard method for detection and subtype differentiation. A review of the *Blastocystis* literature so far suggests that detection and differentiation has not yet been harmonized [22]. The findings of the present study showed that qPCR is a suitable tool for the highly sensitive detection of *Blastocystis* sp., and the NGS approach enables accurate assessment of subtype diversity, in particular, mixed subtype colonization. We believe that the combination of these two approaches will be beneficial for future epidemiological surveys and surveillance studies on *Blastocystis*.

## Supplementary materials

The Supplementary materials of this article are available at https://www.parasite-journal.org/10.1051/parasite/2022029/olm. *Supplementary data 1*: Detailed description of the next-generation sequencing protocol for *Blastocystis.* *Supplementary data 2*: Quantification curve used in qPCR diagnostic protocol for evaluation of the fecal *Blastocystis* load in human DNA samples (in LightCycler LC 480 I; Roche, Basel, Switzerland). The curve was set in the range of 100 to 105 cells per 1 qPCR reaction based on the *Blastocystis* ST3 culture.
