# Supplementary material for: Comparison of molecular diagnostic approaches for the detection and differentiation of the intestinal protist Blastocystis sp. in humans
Source: Parasite. 2022 May 31;29:30. doi: 10.1051/parasite/2022029 (PMC9153396; doi:10.1051/parasite/2022029)
Supplement: Supplementary file 1 — Supplementary data 1: Detailed description of the next-generation sequencing protocol for Blastocystis. [file parasite-29-30-s1.pdf]

Comparison of molecular diagnostic approaches for the detection and differentiation of the intestinal protist *Blastocystis* sp. in humans

Martina Šloufová[1,2], Zuzana Lhotská[1,2], Milan Jirků[1], Klára J. Petrželková[1,3], C. Rune Stensvold[4], Ondřej Cinek[5], Kateřina Jirků Pomajbíková[1,2*]

SUPPLEMENTARY DATA 1: Detailed description of the next-generation sequencing protocol for *Blastocystis.*

Positive samples from qPCR were subjected to NGS to determine *Blastocystis* subtypes according to the method by Maloney et al. (2019). Briefly, amplicons of an informative region (~450 bp) of the SSU rDNA gene were generated using overhang primers, purified, and provisioned with indices and sequencing adaptors using a limited number of PCR cycles with combinatorial indices (Nextera XT Index Kit v2 Set A and D, Illumina, San Diego, CA, USA). The amplicon libraries were purified and equalized using on the SequalPrep plates (Thermo, Waltham, MA, USA), pooled, supplemented with 20% PhiX control to balance the amplicon signal, and sequenced on a MiSeq instrument with the Reagent Kit v2, 2x250 bp (Illumina). The ensuing sequences were downloaded from BaseSpace as demultiplexed fastq files, and processed using the USEARCH10 program (Edgar et al. 2010): primers were trimmed, reads were filtered for quality, and unique sequences defined as zero-radius operational taxonomic units, denoised, their frequencies were tabulated, off-target amplicons were removed and subtypes of *Blastocystis* identified by clustering with a reference set of representative sequences as described in Cinek et al. (2021).

References:

Maloney JG, Molokin A, Santin M. 2019. Next generation amplicon sequencing improves detection of Blastocystis mixed subtype infections. Infection, Genetics and Evolution, 73,119–125.

Edgar RC. 2010. Search and clustering orders of magnitude faster than BLAST. Bioinformatics, 26, 2460-1.

Cinek O, Polačková K, Odeh R, Alassaf A, Kramná L, Ibekwe MAU, Majaliwa ES, Ahmadov G, Elmahi BME, Mekki H, Oikarinen S, Lebl J, Abdullah MA. 2021. Blastocystis in the faeces of children from six distant countries: prevalence, quantity, subtypes and the relation to the gut bacteriome. Parasites and Vectors, 14, 399.
