# Supplementary material for: Comparison of molecular diagnostic approaches for the detection and differentiation of the intestinal protist Blastocystis sp. in humans
Source: Parasite. 2022 May 31;29:30. doi: 10.1051/parasite/2022029 (PMC9153396; doi:10.1051/parasite/2022029)

SUPPLEMENTARY DATA 2: Quantification curve used in qPCR diagnostic protocol for evaluation of the fecal *Blastocystis* load in human DNA samples (in LightCycler LC 480 I; Roche, Basel, Switzerland). The curve was set in the range of 10^0 to 10^5 cells per 1 qPCR reaction based on the *Blastocystis* ST3 culture.

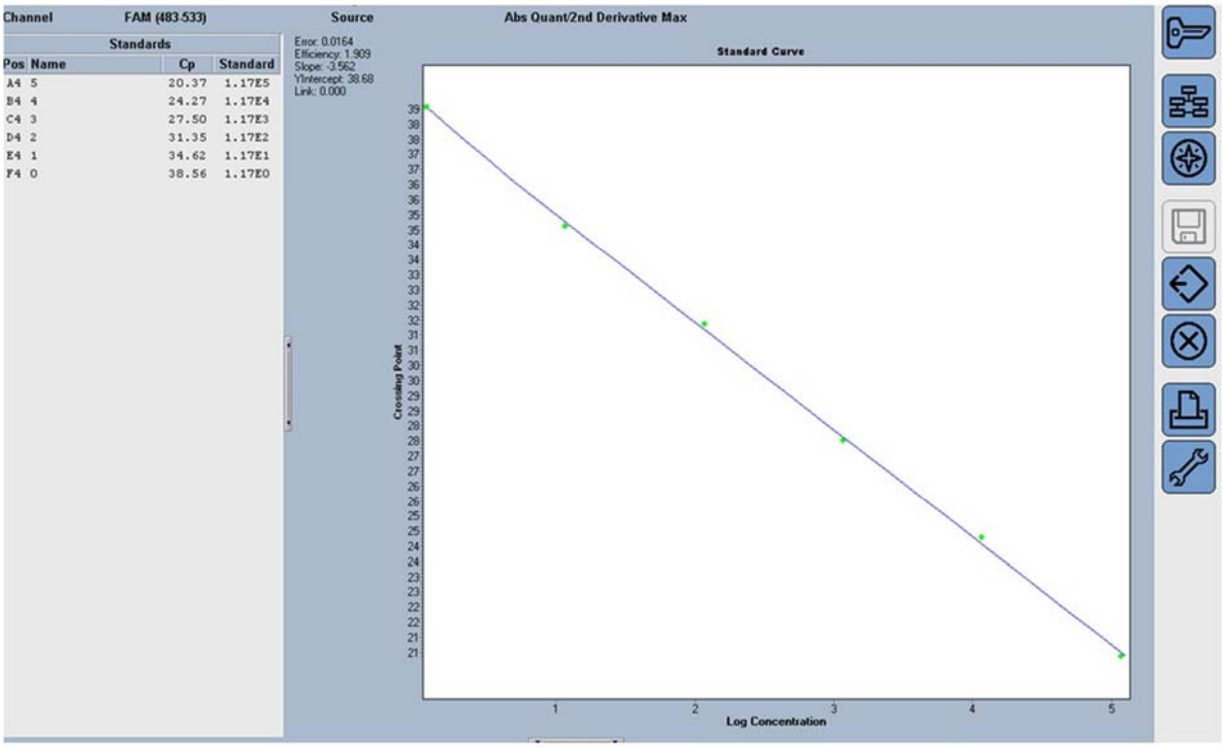

Supplement: Supplementary file 2 — Supplementary data 2: Quantification curve used in qPCR diagnostic protocol for evaluation of the fecal Blastocystis load in human DNA samples (in LightCycler LC 480 I; Roche, Basel, Switzerland). The curve was set in the range of 100 to 105 cells per 1 qPCR reaction based on the Blastocystis ST3 culture. [file parasite-29-30-s2.pdf]
